# Intelligence

**Published:** 1828-02

**Authors:** 


### INTELLIGENCE.

### MONTHLY REPORT OF PREVALENT DISEASES.

The extraordinary mildness of the season has rendered the diseases of the present winter like those of spring or autumn. The constant rain, and consequent damp state of the atmosphere, has rendered Chronic Rheumatism very prevalent; but there has been less than usual of Acute Inflammation; and the Winter Coughs of elderly persons, which during a severe season so frequently prove fatal, have been in general very mild. The popular opinion is that such winters are unhealthy, but we believe this to be a mistaken idea.

### BILLS OF MORTALITY FOR 1827.

### DISEASES.

| | |
|---|---|
| Abscesses | 84 |
| Age, and Debility | 1724 |
| Apoplexy | 417 |
| Asthma | 955 |
| Bedridden | 2 |
| Bile | 8 |
| Cancer | 115 |
| Childbirth | 270 |
| Consumption | 5372 |
| Contraction of the Heart | 1 |
| Convulsions | 2645 |
| Cow-Pox | 1 |
| Croup | 124 |
| Diabetes | 3 |
| Diarrhœa | 16 |
| Dropsy | 1075 |
| Dropsy on the Brain | 763 |
| Dropsy on the Chest | 65 |
| Dysentery | 13 |
| Enlargement of the Heart | 17 |
| Epilepsy | 29 |
| Eruptive Diseases | 24 |
| Erysipelas | 20 |
| Fever | 755 |
| Fever, Typhus | 92 |
| Fever, Intermittent, or Ague | 11 |
| Fistula | 4 |
| Flux | 11 |
| Gout | 40 |
| Hemorrhage | 51 |
| Hernia | 46 |
| Hooping Cough | 767 |
| Hydrophobia | 1 |
| Inflammation | 2356 |
| Inflammation of the Liver | 136 |
| Insanity | 273 |
| Jaundice | 43 |
| Jaw locked | 3 |
| Measles | 525 |
| Miscarriage | 2 |
| Mortification | 345 |
| Ossification of the Heart | 9 |
| Palpitation of the Heart | 18 |
| Palsy | 34 |
| Paralytic | 197 |
| Pleurisy | 24 |
| Rheumatism | 38 |
| Scrofula | 9 |
| Small-Pox | 616 |
| Sore Throat, or Quinsey | 16 |
| Spasm | 55 |
| Stillborn | 936 |
| Stone | 23 |
| Stoppage in the Stomach | 22 |
| St. Vitus's Dance | 1 |
| Suddenly | 107 |
| Teething | 503 |
| Thrush | 72 |
| Tumor | 21 |
| Venereal | 4 |
| Worms | 2 |
| Total of Diseases | 21911 |

### CASUALTIES.

| | |
|---|---|
| Broken Limbs | 2 |
| Burnt | 36 |
| Drowned | 134 |
| Excessive Drinking | 5 |
| Executed | 6 |
| Found dead | 17 |
| Fractured | 1 |
| Frighted | 1 |
| Frozen | 1 |
| Killed by Falls and several other accidents | 106 |
| Killed by Fighting | 3 |
| Murdered | 3 |
| Poisoned | 3 |
| Scalded | 7 |
| Starved | 6 |
| Strangled | 2 |
| Suffocated | 4 |
| Suicides | 44 |
| Total of Casualties | 381 |

Christened....Males, 15,205—Females, 14,720—In all, 29,925.
Buried ......Males, 11,296—Females, 10,996—In all, 22,292:

Whereof have died—

| | |
|---|---|
| Under Two Years of Age | 6580 |
| Between Two and Five | 1875 |
| Five and Ten | 850 |
| Ten and Twenty | 862 |
| Twenty and Thirty | 1565 |
| Thirty and Forty | 1831 |
| Forty and Fifty | 2134 |
| Fifty and Sixty | 2128 |
| Sixty and Seventy | 2044 |
| Seventy and Eighty | 1680 |
| Eighty and Ninety | 666 |
| Ninety and a Hundred | 74 |
| A Hundred | 1 |
| A Hundred-and-One | 1 |
| A Hundred-and-Two | 1 |

Increased in the Burials this year, 1534;—arising principally from two years being included in the return from St. Leonard, Shoreditch.

☞ There have been executed within the Bills of Mortality, 19; of which number only 6 have been reported as such.

---

### *Royal College of Surgeons in London.*

*Regulations of the Council relating to the Age and Professional Education of Candidates for the Diploma of the College.*—I. The only schools of anatomy and physiology recognised, are London, Dublin, Edinburgh, Glasgow, and Aberdeen.

II. Attendance upon the surgical practice of an hospital will be recognised, provided such hospital contain at least 100 patients.

III. No person under twenty-two years of age shall be admitted a member of the College.

IV. The following certificates will be required of candidates for the diploma of the College:—1. Of having been engaged six years, at least, in the acquisition of professional knowledge. 2. Of having regularly attended three or more winter courses of anatomy and physiology, and two or more winter courses of dissections and demonstrations, delivered at subsequent periods. (Two courses of anatomy and physiology in Edinburgh or Dublin, which are of six months' duration, and the accompanying courses of dissections and demonstrations, will be considered as equivalent to the foregoing attendance.) 3. Of having regularly attended two or more courses of lectures on the principles and practice of surgery; one of which shall have been delivered in a recognised school of anatomy. 4. Of having also attended the following lectures, viz. Two courses on the theory and practice of physic of three months each, or one of six months; one course on materia medica and botany: two courses on chemistry of three months each, or one of six months; two courses on midwifery of three months each, or one of six months. 5. And of having attended, during the term of at least one year, the surgical practice of one or more of the following hospitals: viz. St. Bartholomew's, St. Thomas's, the Westminster, Guy's, St. George's, the London, and the Middlesex, in London; the Richmond, Steeven's, and the Meath, in Dublin; and the Royal Infirmaries, in Edinburgh, Glasgow, and Aberdeen;—or, during four years, the surgical practice of a recognised provincial hospital, and, six months at least, the practice of one of the above-named hospitals in the schools of anatomy.

V. Candidates under the following circumstances, of the required age, and who have been engaged five years in the acquisition of professional knowledge, will be admissible to examination, viz. Members, or licentiates in surgery, of any of the legally constituted Colleges of Surgeons in the united kingdom; and graduates in medicine of any of the Universities in the united kingdom, provided they have attended lectures, the practice of an hospital, and performed dissections, as required in Regulation IV.

VI. The required certificates shall express the dates of the commencement and of the termination of attendance on each course of lectures, and dissections: and also of attendance on hospital practice.

VII. The required certificates shall be delivered at the College ten days before candidates can be admitted to examination. By order,

*5th day of January*, 1828. EDMUND BELFOUR, Sec.

---

*The London and Southwark Midwifery Institution.*—This charity was established some years ago, for the purpose of affording professional assistance to poor women at their own houses during confinement in childbed; since which time it has been in active operation, and has hitherto been supported by the exertions of a few individuals. The number of applicants has, however, so greatly increased of late, (upwards of 400 having been relieved during the last twelve months,) that it was quite impossible to carry it on in the same private manner without materially circumscribing its beneficial operations; and hence the original founders of the institution, encouraged as they were by promises of support from a highly respectable number of individuals, have invited the co-operation of the public, in order that they might be enabled to extend its benefits. The following gentlemen have been appointed the medical officers; and, as we understand that such an institution is much required in the district which it embraces, we hope that it will meet with the encouragement it appears so well to merit.

*Consulting Physicians.*—Dr. OVEREND, 60, Old Broad-street, Physician to the Islington Dispensary: Dr. ROBERTS, 32, New Bridge street, Physician to the Seamen's Hospital, and senior Physician to the South London Dispensary.

*Consulting Accoucheurs.*—CHARLES WALLER, Esq. 93, Bartholomew Close, M.R.C.S. and Lecturer on Midwifery and the Diseases of Women and Children: EDWARD DOUBLEDAY, Esq. 10, Great Surrey-street, M.R.C.S. &c.

### METEOROLOGICAL JOURNAL,

*From December 20th,* 1827, *to January 20th,* 1828.

By Messrs. HARRIS and Co. Mathematical Instrument Makers, 50, High Holborn.

| December | Rain gauge. | Moon. | Thermom. | | | Barometer. | | De Luc's Hygrom. | | Winds. | | Atmospheric Variations. | | |
|---|---|---|---|---|---|---|---|---|---|---|---|---|---|---|
| | | | 9 A.M. | MAX. | MIN. | 9 A.M. | 10P.M. | 9A. M. | 10P.M. | 9 A.M. | 10P.M. | 9 *a.m.* | 2 *p.m.* | 10 *p.m.* |
| 20 | | | 46 | 49 | 39 | 29.43 | 29.45 | 90 | 90 | W | WSW | Fair | Fair | Cloudy |
| 21 | | | 41 | 49 | 46 | 29.58 | 29.34 | 93 | 98 | W | S | — | — | — |
| 22 | | | 48 | 53 | 45 | 29.28 | 29.40 | 92 | 98 | W | WSW | Cloudy | — | Rain |
| 23 | | | 46 | 55 | 42 | 29.81 | 29.93 | 90 | 90 | N | N | — | — | Fair |
| 24 | | | 44 | 56 | 41 | 29.93 | 29.97 | 90 | 87 | W | WNW | Rain | Rain | Fine |
| 25 | | ☽ | 42 | 50 | 45 | 30.17 | 30.28 | 87 | 94 | W | W | Fine | Fine | Cloudy |
| 26 | | | 48 | 52 | 42 | 30.32 | 30.34 | 97 | 96 | W | WSW | Fair | Fair | — |
| 27 | | | 46 | 52 | 38 | 30.36 | 30.44 | 98 | 98 | W | NE | — | — | — |
| 28 | | | 39 | 43 | 35 | 30.53 | 30.48 | 98 | 98 | NE | SSW | Cloudy | — | — |
| 29 | | | 36 | 38 | 31 | 30.40 | 30.27 | 98 | 93 | WSW | SSE | Foggy | Foggy | Foggy |
| 30 | | | 36 | 43 | 36 | 30.38 | 30.10 | 96 | 98 | SSW | S | — | — | Fine |
| 31 | | | 42 | 48 | 42 | 29.97 | 29.68 | 98 | 98 | W | SW | — | Fair | — |
| Jan. | | | | | | | | | | | | | | |
| 1 | | | 45 | 47 | 36 | 29.48 | 29.60 | 98 | 98 | NNE | N | Fair | — | Fair |
| 2 | | ○ | 39 | 50 | 45 | 29.77 | 29.41 | 98 | 98 | N | SW | — | Rain | Cloudy |
| 3 | | | 46 | 50 | 34 | 29.56 | 29.60 | 98 | 90 | NW | WNW | Cloudy | Fair | — |
| 4 | | | 36 | 44 | 32 | 29.73 | 29.52 | 97 | 95 | W | NW | — | Rain | — |
| 5 | | | 34 | 35 | 34 | 29.52 | 29.53 | 96 | 98 | W | NNW | — | Snow | Fair |
| 6 | | | 36 | 39 | 33 | 29.54 | 29.81 | 96 | 98 | W | SE | — | Cloudy | Cloudy |
| 7 | | | 35 | 36 | 33 | 29.86 | 29.74 | 89 | 92 | SE | E | — | — | — |
| 8 | | | 34 | 38 | 33 | 29.75 | 29.85 | 85 | 90 | ESE | NE | — | — | — |
| 9 | | | 34 | 35 | 29 | 29.85 | 29.77 | 87 | 87 | SE | E | — | — | — |
| 10 | | ☾ | 31 | 34 | 28 | 29.57 | 29.52 | 85 | 92 | N | SE | — | — | — |
| 11 | | | 32 | 40 | 36 | 29.41 | 29.47 | 99 | 99 | ESE | SE | Snow | Sleet | Fog |
| 12 | | | 38 | 46 | 42 | 29.63 | 29.51 | 99 | 99 | WSW | SE | Fog | Rain | Rain |
| 13 | | | 44 | 47 | 38 | 29.45 | 29.45 | 99 | 94 | W | W | Cloudy | Cloudy | Cloudy |
| 14 | | | 43 | 45 | 35 | 29.37 | 29.40 | 99 | 98 | NW | NE | — | — | Rain |
| 15 | | | 35 | 37 | 31 | 29.48 | 29.61 | 99 | 98 | NE | NE | — | Rain | Snow |
| 16 | | ● | 38 | 40 | 38 | 29.68 | 29.68 | 99 | 98 | NE | SE | — | — | Rain |
| 17 | | | 46 | 50 | 49 | 29.68 | 29.93 | 96 | 94 | SW | SW | — | — | Cloudy |
| 18 | | | 54 | 56 | 46 | 30.04 | 30.06 | 98 | 89 | SW | WSW | — | Cloudy | Fair |
| 19 | | | 49 | 55 | 47 | 30.13 | 30.13 | 86 | 90 | WSW | SW | Fair | Fine | — |

